# Monitoring of patients with active inflammatory bowel disease

**DOI:** 10.3389/fgstr.2023.1172318

**Published:** 2023-05-25

**Authors:** T. Kucharzik, B. Verstockt, C. Maaser

**Affiliations:** ^1^ Department of Internal Medicine and Gastroenterology, Klinikum Lüneburg, Lüneburg, Germany; ^2^ Department Gastroenterology and Hepatology, University Hospitals, KU Leuven, Leuven, Belgium; ^3^ Department of Chronic Diseases and Metabolism, KU Leuven, Leuven, Belgium; ^4^ Inflammatory Bowel Disease Outpatient Department, Department of Geriatriec, Klinikum Lüneburg, Lüneburg, Germany

**Keywords:** monitoring, intestinal ultrasound, inflammatory bowel disease (IBD), magnetic resonance enterography (MRE), biomarker, transmural healing, mucosal healing (MH), endoscopic remission (ER)

## Abstract

In the current treat-to-target era, close and tight monitoring of patients with inflammatory bowel disease has become increasingly important. Although the importance of patient reported outcomes (PROMs) cannot be underestimated, its moderate association with biochemical and histo-endoscopic outcomes highlights the need for additional monitoring strategies. Endoscopic and histological remission are linked with improved long-term outcomes, but require more invasive assessments. Hence, non-invasive monitoring modalities are becoming increasingly relevant, with emerging evidence demonstrating the added clinical value of transmural assessment, both in Crohn’s disease and ulcerative colitis. The current review covers the multiple treatment targets present in IBD care, and focusses in particular on the increasing importance of intestinal ultrasound. Finally, we propose a potential algorithm to monitor patients with IBD in daily clinical practice and highlight gaps for future research in monitoring IBD strategies.

## Introduction

Treatment targets of inflammatory bowel disease (IBD) have evolved over the last decade. Therapy goals consisting of symptom control have shifted to control of disease activity with endoscopic remission (ER) or even further. Beyond the achievement of mucosal healing (MH) it needs to be considered that Crohn’s disease (CD), as well as ulcerative colitis (UC), involve transmural inflammation that cannot be fully appreciated with endoscopy. Therefore, transmural assessment of disease activity by cross-sectional imaging with magnetic resonance enterography (MRE) and intestinal ultrasound (IUS) have been implemented to assess disease control. For UC histologic disease control has emerged as new treatment goal during the last couple of years.

The current non-systematic review will summarize different clinical and objective parameters for the use of monitoring patients with active IBD. The following keywords have been used: “monitoring”, “inflammatory bowel disease”, “ulcerative colitis”, “Crohn’s disease”, “intestinal ultrasound”, “biomarker”, “CRP”, “faecal calprotectin”, “endoscopy”, “MRE”, “transmural healing”, “mucosal healing”. The literature search has been performed by one of the authors (TK) and cross-checked by the co-authors (CM and BV). Relevant literature between 2000 and march 2023 has been searched in PubMed, Embase and Cochrane database in addition to own files. The final manuscript was critically reviewed by all authors.

The potential use of IUS and its combination with other surrogate markers of inflammation in follow up of patients with IBD will be specifically highlighted.

## Clinical parameters for monitoring IBD

Symptoms reflect early disease experience and quality of life. Therefore, symptom control will always be important to patients. When considering symptoms, patient related outcomes (PRO)s are becoming the standard of measure. PROs strongly correlate with well-being and should therefore be frequently assessed during disease course. In patients with CD, the most commonly used PRO is the PRO2 which is a sum of the stool frequency and abdominal pain items from the CDAI (1). In UC, PRO2 which is composed of stool frequency and rectal bleeding, has become the current standard of assessing symptoms. Unlike CD, clinical symptoms in UC correlate well with endoscopic disease activity, with absence of diarrhea and rectal bleeding being an independent predictor of long term clinical outcomes (2). Most recently, urgency has been established as relevant parameter for disease activity in patients with UC (3, 4).

IBD has an enormous impact on the mental and emotional well-being of patients. A patient-centric clinical care model has therefore recently been suggested in order to achieve holistic remission (5). General measures of quality of life and the functional status of the IBD patient can be evaluated by questionaires or validated tools such as the IBD disability index or the IBD disk (6, 7).

Recent STRIDE-II criteria consequently demand for symptom control as initial treatment goal (8). However, it is also well-known that, in particular in CD patients, there may be a discordance between symptoms and intestinal inflammation, and therefore treatment decisions focused solely on symptom control may result in over- or undertreatment (9, 10). Persistent subclinical inflammation may result in progressive structural damage and potentially complications in CD, but also in UC (11). It is also well known that patients, who achieve clinical remission as defined by activity indices such as CDAI, may not achieve CRP normalization and/or endoscopic remission. This has previously been demonstrated for steroids, as well as other therapeutic agents (12, 13). Discordance between symptoms and objective markers have also been made in the SONIC trial, where at least half of the patients treated with a combination of infliximab plus azathioprine and who were in complete clinical remission still had endoscopic and/or biochemical evidence of residual inflammation (14). In contrast, other patients with endoscopic remission and CRP normalization had persistent symptoms, presumably linked to an associated functional pathophysiology. Similarly in UC, the correlation between PROs and histo-endoscopic outcomes is far from perfect (15). Although the value and importance of patient reported outcome measures (PROMS) in CD and UC is well recognized (16, 17), PROMS cannot be used as sole therapeutic targets and objective measures of inflammation need to be added (8).

## Role of biomarkers

Non-invasive biomarkers are increasingly used in the tight control model of intestinal inflammation in IBD. The use of biomarkers enables determination of disease activity and disease risk stratification. Targeted monitoring at defined time points to assess outcomes in response to therapy has been shown to allow for quick therapeutic adjustments before chronic bowel damage may occur (18).

### Relevance of CRP

CRP has been widely used to monitor patients with CD and UC. Even though CRP is used as serum biomarker to follow-up disease activity, in clinical practice several limitations of CRP need to be considered in addition to the fact that CRP is neither bowel nor disease specific. In CD, up to 20% of patients who have active (ileal) disease will not have an elevated CRP (19, 20). CRP levels only modestly correlate with endoscopic disease activity in UC, and CRP levels are usually much lower compared to active CD patients with more frequent false negative results in UC compared to CD (21). Despite the well-known limitations, CRP is still worldwide used as serum marker for measuring IBD activity, and has been shown that timely measured CRP during treatment is able to predict response to treatment and has been shown to be useful in follow-up of IBD patients with active disease in CD as well as in patients with UC (20). Normalization of CRP at 8-14 weeks after treatment with anti-TNF predicts remission at 1 year (22–24). Similar results could be obtained in *post hoc* analysis of the ACCENT trial with a 60% decrease of CRP at week 14 (25).

### Faecal calprotectin and its role in monitoring IBD

Non-invasive surrogate markers for intestinal inflammation are increasingly used to determine intestinal inflammation and to follow up patients after treatment initiation. Faecal calprotectin (FC) has been emerged as the most popular stool marker and has proven to be an objective marker of intestinal inflammation in CD as well as in UC (26, 27). Various studies have shown that FC correlates better with endoscopic disease activity than the symptom based indices (27–30). In both diseases, FC has been shown to be better than CRP as surrogate marker for endoscopic disease activity (31). Differences in the use of FC in assessing endoscopic disease activity between UC and CD have been determined as well and FC has been shown to be a better endoscopic disease activity marker in UC compared to CD (27). FC also appears to be highly effective to detect endoscopic ulcerations in CD regardless of location but requires a lower cut-off value in patients with pure ileal involvement (32, 33). FC has also shown to have disadvantages with regard to assessing the extent of inflammation (26) and it has been shown to be less useful in proctitis (34).

Even though several studies have shown that FC nicely correlates with individual disease activity, the optimal cut-off value for FC still needs to be defined (35). The most widely used cut-off value below 250 µg/mg indicates endoscopic remission in patients with IBD (36). More recent studies suggest that lower FC levels might favourite to correlate with achieving histologic or transmural remission (8). Thresholds for FC for differentiating histologic remission and activity in UC vary between 40 to 250 µg/mg (37, 38). In monitoring disease course, decreasing levels of FC nicely correlate with clinical response and may predict sustained remission (39, 40). In contrast, repeated FC values increasing the normal rate show up to 83% probability of developing disease relapse within the next three months in patients with asymptomatic IBD (36). Faecal calprotectin determined in patients with CD at week 12 to 14 after anti-TNF initiation predicts clinical remission, with cut off-values between 80-170 µg/mg (41, 42). In another study, anti-TNF induced FC decrease of 50% at week 12 was associated with corticosteroid-free remission at 1 year (22). In patients with UC, FC at a level of 168 µg/mg after treatment induction is associated with 79% sensitivity and 57% specificity for predicting endoscopic healing at 1 year (42, 43).

The optimal monitoring interval of FC in follow up of active IBD patients, as well as in asymptomatic patients in UC as well as CD, is still under debate (44). Based on available data, the recent ECCO-ESGAR diagnostic guideline suggests to assess FC every 3-6 months depending on remission duration and on current therapy (45–47). Further scientific evaluation is required if there is an advantage of shorter testing intervals. It needs to be evaluated if home-tests for FC which are more frequently offered, may reduce delays in clinical decision making and treatment adjustments.

Preliminary data have shown that combining serum and stool biomarkers may increase the sensitivity to determine disease activity, and may improve outcome prediction better than the individual use of single biomarkers. A combination of CRP and FC was superior in the CALM trial to FC alone in predicting endoscopic remission after treatment with adalimumab in CD patients (18). As a combination of elevated CRP and fCalpro may predict relapse in asymptomatic patients its use may be helpful to guide treatment de-escalation and exit strategies in clinical practice (48).

It needs to be defined if combination of different biomarkers such as FC plus CRP plus IUS offers additional benefit for monitoring disease activity in individual patients.

## Endoscopy

### Relevance of mucosal healing

Endoscopic disease control is a well-established treatment goal, but associated with inconsistent definitions of ER and MH in the literature. As ER is mainly used for endoscopic evaluation and MH involves endoscopic remission combined with histologic remission (8, 49), we here use the term ER for consistency. ER is best defined as an absence of ulcers in CD, which has been shown to predict the likelihood of clinical relapse, the risk of surgery as well as the risk of hospitalisation (50, 51). In a treat-to-target strategy in CD, ER has become the therapeutic goal as just recently defined by STRIDE-II criteria (8). Remission here includes steroid-free patient reported outcome remission, as well as ER defined as resolution of ulceration determined by ileocolonoscopy. The relevance of ER in UC has been determined in several studies. In a recent meta-analysis, patients with UC in clinical remission who achieved an endoscopic Mayo score (MES) 0 had a 52% lower risk of relapse compared with patients with MES 1 (52).

Even though several studies clearly demonstrate that patients achieving more rigorous treatment endpoints have a lower risk of clinical relapse than patients with only the conventional definition of clinical remission, prospective RCTs to use ER as treatment target are still lacking.

The recent STARDUST trial showed that timely escalation of ustekinumab therapy for patients with CD, based on early endoscopic response, clinical symptoms, and biomarkers, did not result in significantly better endoscopic outcomes at week 48 than symptom-driven decisions alone (53). Even though in a recent *post-hoc* analysis from STARDUST after 2 years a difference in composite endpoints of disease complications could be determined (54). The REACT 2 trial compared clinical outcome for treatment of patients with CD based on endoscopy results with treatment based on clinical parameters. The primary endpoint was not reached. However, sub-analysis could demonstrate that patients with an elevated CRP and mucosal ulcerations benefit from the treat to target approach (55).

The best time point to evaluate ER in Crohn’s disease is not clearly defined yet and probably depends on different factors. As most studies have determined ER at least six months after treatment initiation, the recent ECCO/ESGAR diagnostic guideline suggests to evaluate ER in CD approximately 6 months after treatment initiation (45).

In UC, ER might be determined earlier. The current ECCO/ESGAR diagnostic guideline suggests to evaluate ER 3-6 months after treatment initiation, keeping in mind that the potential to induce ER varies between different therapeutic agents (45).

As CD is a transmural disease, there might be limitations of the existing target of ER as intestinal damage may currently persist despite the presence of ER (56). In a recent prospective study of children with CD, one third of patients had healing of the mucosa but no transmural healing (TH) (57). In another study on paediatric CD patients more than 25% of patients with endoscopic remission showed persistent signs of transmural inflammation (58).

### Relevance of histology

The notion of MH has recently been evolved from an endoscopic-based definition to a composite of endoscopy and histopathology. Various drugs such as ustekinumab, filgotinib, upadacitinib and ozanimod have recently been approved for use in patients with UC and have been evaluated and achieved a label for both endoscopic and histologic remission as trial end points based on a definition of “histoendoscopic mucosal healing,” defined as both endoscopic and histologic improvement (59–62). Multiple observational studies have suggested that patients with UC who achieve endoscopic remission (MES 0) or histologic remission, or both, may have a lower risk of clinical relapse and disease-related complications than those who achieve conventionally defined remission.

In a systematic review and meta-analysis of 31 studies that included 2608 patients with UC in clinical remission among patients with MES 0, those who achieved histologic remission had a 63% lower risk of relapse, compared with patients with histologic activity (52). An estimated clinical relapse risk of only 5% per year was observed in this patient population, compared with 13.7% for those with endoscopic remission only. The more rigorous remission target was associated with a substantially better prognosis. Another meta-analysis that included 28 studies, confirmed that patients with ER but persistent histologic disease activity had a higher risk of clinical relapse (63). A reduction of clinical relapse of about 58% could be determined in this meta-analysis in patients with histologic remission compared to patients with UC with histologic activity. One of the main problems with the studies is the heterogeneous definition of histologic activity. Various histologic activity scores are available and current studies used validated and non-validated histologic disease activity indices with different cut-offs which complicates interpretation of the results (64). Prospective controlled trials that determine the efficacy of current therapies to achieve such stringent endpoints and to prove general feasibility and cost-effectiveness of such strategies are ongoing. The ongoing multicentre, randomised, controlled VERDICT trial is to determine whether a treatment target of corticosteroid (CS)-free symptomatic + endoscopic + histologic remission is superior to CS-free symptomatic remission alone in moderately to severely active UC. As long as the results of those studies are not available, histologic healing should not be regarded as therapeutic target in clinical practice. In this line, the recent STRIDE II criteria define histologic remission currently not as a formal treatment target, but rather as a criterium which is associated with a good prognosis (8). The concept of “disease clearance” in UC aims to achieve clinical and biological remission as well as mucosal healing (endoscopic, histological, and in potentially molecular) in these patients (65).

Few data on the relevance of histologic activity in CD demonstrate advantage of histologic remission over endoscopic remission only (66). However, these data are scarce, most probably because of the transmural nature of disease in CD.

## Use of cross-sectional imaging for monitoring IBD

As therapeutic targets in Crohn’s disease have shifted from targeting symptoms towards reducing objective inflammatory activity, frequent monitoring of disease activity is required to adjust therapy. As endoscopic assessment of mucosal healing inadequately reflects transmural disease activity, cross-sectional imaging such as a MRE or IUS is required.

### Relevance of transmural remission

Considering the limitations of determining ER during endoscopy, more inclusive transmural remission (TR) may be a more appropriated therapeutic goal in contrast to ER (67, 68). Therefore TR as a resolution of not only mucosal ulceration but also transmural disease related bowel alterations, might represent a more stringent target in routine clinical practice (69–71). In this review TR and TH are used as synonyms. TR as a predictor of long-term outcomes in IBD has been studied by several groups. Several prospective studies could show that patients with TR after biologic therapy, determined by MRE reveal significantly less need for surgery, less need for hospitalisation and less treatment intensification in comparison to no remission, but also compared to patients with MH only (72, 73). A long-term study from a retrospective Spanish cohort could recently demonstrate that MRE determined TR is also associated with a long-term outcome in a follow-up of 5 years with a significant difference between TR and ER (74).

For IUS, several groups prospectively investigated one year outcomes depending on treatment status. Comparable to MRE results, TR as determined by IUS was associated with significantly better long-term outcomes in comparison to no-remission, but also to ER only (71, 75, 76). In these studies, TR was superior to ER for clinical outcomes including need for hospitalisation and for surgery as well as need for treatment escalation.

### MRI/CT

Both MRE or CTE and IUS are equally accurate at detecting small and large bowel disease activity in IBD, and may therefore all be used as monitoring modalities in determining disease activity in IBD (77, 78). However, based on radiation safety, CTs should be avoided to monitor disease activity whenever possible (45). Using MRI, recent consensus statements defined therapeutic response according to changes in imaging parameters of disease activities such as bowel wall thickness and T2 signal (79, 80). MRI parameters for monitoring disease are categorized in four categories: transmural remission (normalization of all features), response (decrease in the severity of extent of imaging findings within an inflamed segment), stable disease (no clear change in severity or extent) or progression (worsening in parameters of inflammation) (80). These categories could be used for MRI, as well as for IUS. In patients with CD, the stringent definition of transmural remission requires complete resolution of all inflammatory, as well as extramural findings, with a normalization of all parameters: bowel wall thickness (BWT) < 3 mm, no signs of hyperperfusion, no edema and no ulcers or fat stranding (80). Less stringent and perhaps more realistic definitions allow the resolution of inflammation, with residual findings such as mild wall thickening or mild hyperperfusion. The definition of TR in MRI therefore may depend on disease duration and existing bowel damage. Established and validated MRI disease activity scores can be used for diagnosis and staging of CD (81, 82). MRI disease activity scores may therefore be used in follow-up and assessing treatment response for scientific purposes, but also in clinical practice (83).

MRI has also been shown to be useful in guiding clinical decisions. In a recent study comparing colonoscopy followed by MRI versus MRI followed by colonoscopy in patients with CD, it could be shown that information from MRI alone was sufficient for guiding therapy in 80% of cases, whereas information from colonoscopy alone was sufficient in only 34%. In another study comparing point-of-care IUS with MRI in patients with CD, both modalities had a high impact on clinical decision making and changes to management resulting from IUS and MRI were highly concordant (84).

### Intestinal ultrasound

**Figure 1:**
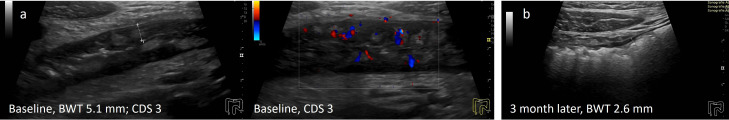
Treatment response in CD determined by IUS **(A)** Before treatment; **(B)** Transmural remission 3 months after treatment with infliximab. 29y old male patient with symptomatic colonic CD (L2B1). Normalization of BWT and CDS 3 months after initiation of infliximab.

The main advantages of IUS over other cross sectional imaging modalities such as MRI and CT are its non-invasiveness, rapid availability, no requirement of preparation, no radiation, its fast results and low costs. IUS is patient-centered and patients prefer IUS over other more invasive diagnostic modalities for monitoring disease activity (85). Intestinal ultrasound has been shown to directly reflect transmural disease activity and can be determined on-site by simple parameters such as bowel wall thickness, vascularization, echostratification and inflammatory fat assessment (86, 87). If bowel thickness is done under standardized conditions, the interrater variability is very low. In a recent study the ICC for bowel thickness was almost perfect with 0.96 (88). In CD, the relevance of transmural remission has recently been shown in the multicenter TRUST-CD trial where a significant proportion of patient revealed transmural response and TR already 3 months after treatment induction (89, 90). Early transmural response in patients with CD treated with ustekinumab as determined by central read IUS, could then be detected as soon as four weeks after treatment initiation (91). Transmural response in patients with CD may even occur earlier but this has not been determined in a systematic manner yet. TR may take longer, depending on the choice of treatment and appears to occur faster in the colon compared to the terminal ileum (**Figure 1**). In the STARDUST-IUS study, it could also be shown that in bio-naive patients TR and transmural response occurs earlier compared to bio-experienced patients. As already discussed in the previous section, TR induced by IUS is associated with lower risk of bowel damage progression compared to EH and with a better long-term outcome. In this context, IUS may also be used as monitoring tool to predict hospitalization, surgery or even cancer and mortality risk in individual patients which needs to be further evaluated in future studies.

**Figure 2:**
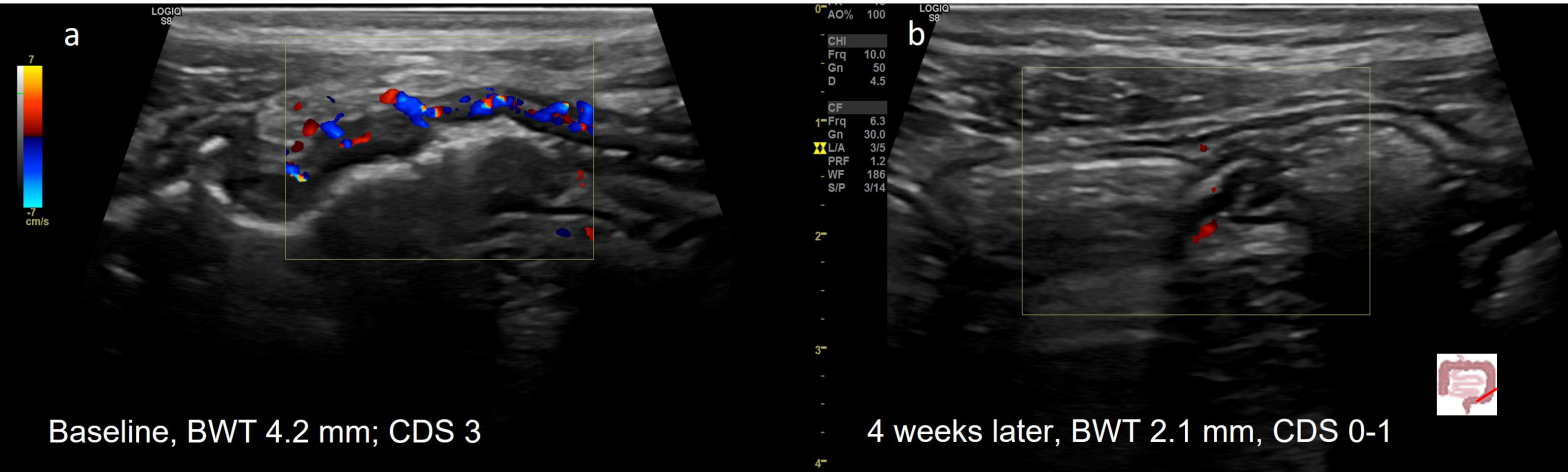
Treatment response in a 20y old patient with UC (E3) determined by IUS **(A)** Before treatment; **(B)** Transmural Remission 4 weeks after treatment with upadacitinib. 20y old male patient with active UC (E3). Normalization of BWT and CDS 4 weeks after initiation of upadacitinib.

Recent studies could demonstrate that IUS also appears to be useful as a monitoring tool in patients with UC. A relevant proportion of patients reveals disease manifestations beyond the mucosa with thickening of the submucosa or even alterations of the extraintestinal tissue indicating that UC presents features of a transmural disease. Relevant disease activity parameters such as bowel wall thickness and vascularization could be normalised in the majority of patients as soon as two weeks after treatment initiation in a recent multicenter trial (92) (**Figure 2**). In patients with acute severe ulcerative colitis, improvement in bowel wall thickness could be observed as soon as 24 - 48 hours after treatment initiation and IUS improvement was predictive for a clinical response in patients with ASUC (93). Rapid improvement of bowel wall thickness in patients with acute UC could also recently be observed in a monocenter study in patients treated with tofacitinib (94). IUS data in this study showed good correlation with endoscopic activity. The submucosa was the most responsive wall layer of treatment response in this study. Disease manifestations in the rectum are usually difficult to detect and to monitor by using transabdominal IUS. Transperineal ultrasonography has recently been shown to accurately determine disease activity of UC in the rectum and to follow up proctitis after treatment initiation (95).

**Figure 3:**
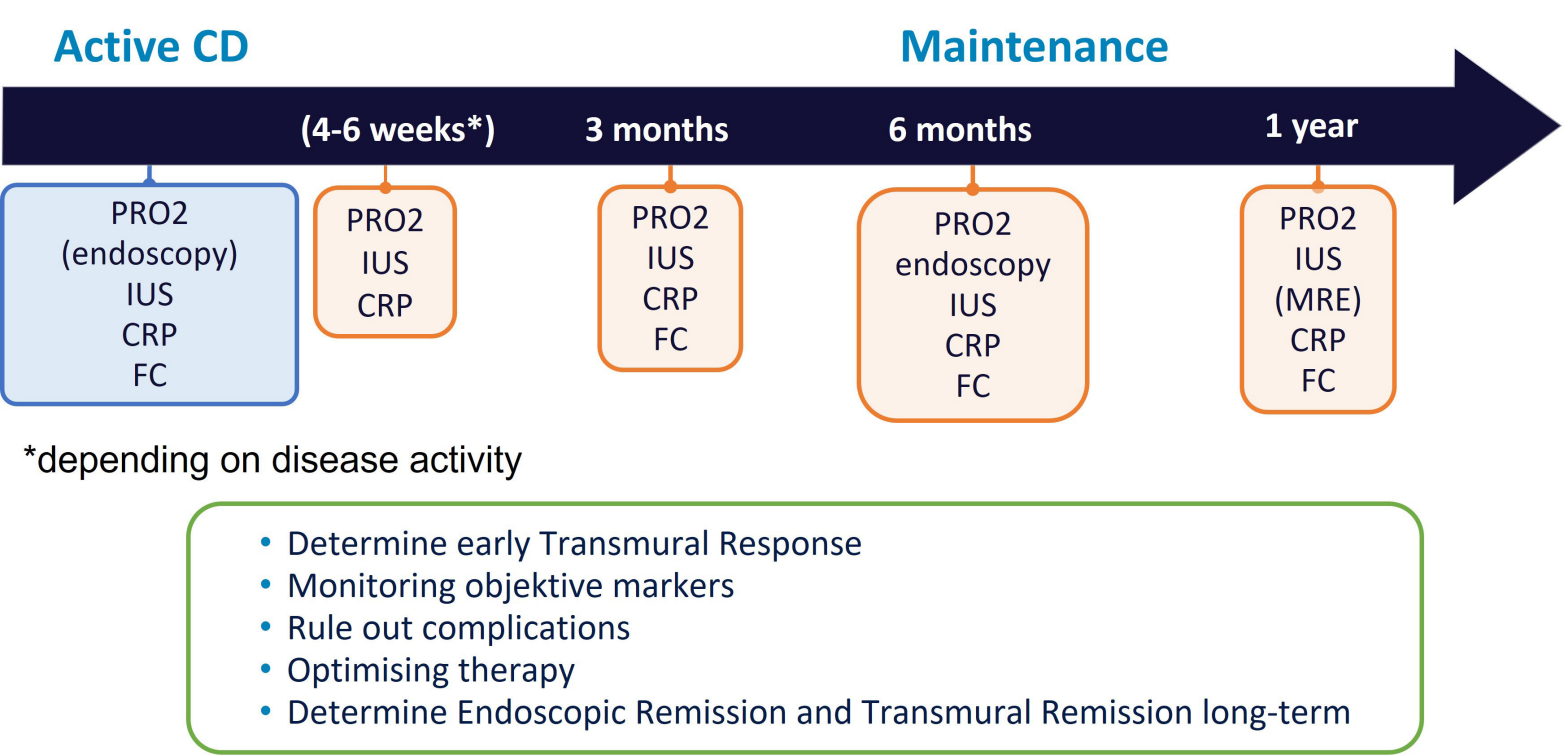
Potential monitoring algorithm for CD.

**Figure 4:**
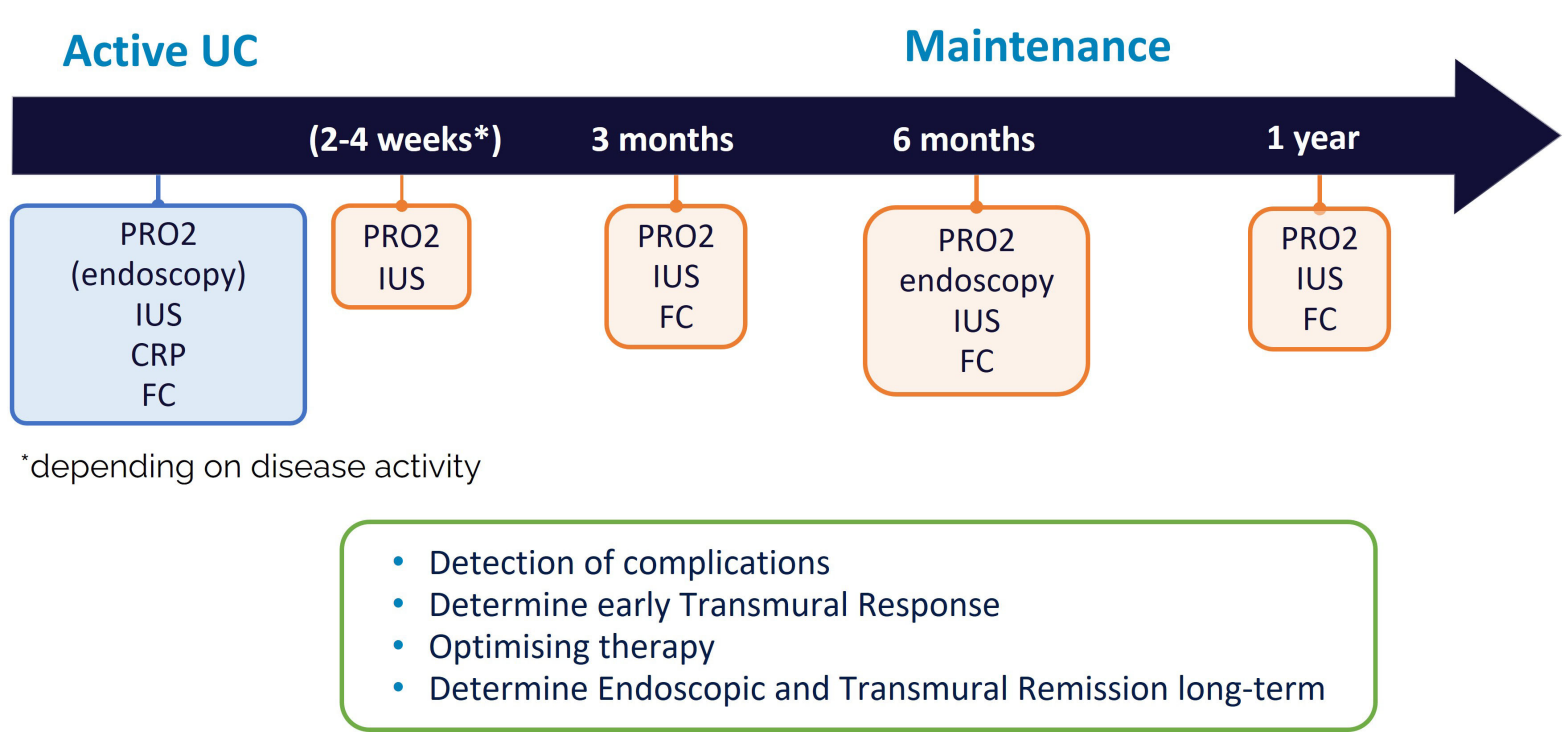
Potential monitoring algorithm for UC.

Even though IUS is increasingly used for monitoring of patients with IBD, validated definitions on transmural response and TR defined by IUS are still lacking. Recent expert consensus proposed parameters on how to define transmural response and TR, as well as on monitoring criteria (86). It has been suggested that response in CD patients should initially be assessed in the small and large bowel at week 14 +/-2 after treatment initiation, regardless of treatment. Early IUS assessment in certain situations may be beneficial between week 4 – 8 (86) (**Figure 3**). Clear timepoints for monitoring for UC are still lacking. As IUS response can be determined at earlier timepoints after treatment initiation (92, 93), early IUS assessment may be beneficial already after 2 – 4 weeks in the majority of patients (**Figure 4**).

### Potential algorithm for monitoring patients with IBD in clinical practice

Current STRIDE-II criteria define three step treatment goals in the management of patients with IBD that include improvement of clinical symptoms, objective biomarkers and endoscopic disease activity (8). Optimal time points to assess the achievement of treatment goals during the monitoring process are less defined, nor are the use of different diagnostic modalities at different time points. Current diagnostic guidelines promote endoscopic evaluation in patients with UC 3-6 months after treatment initiation, and after approximately 6 months in patients with CD (45). Transmural changes in BWT and vascularization as determined by IUS may occur more rapid in patients with UC compared to CD. Early changes in BWT in patients with active UC can be determined by IUS as early as 1-2 days after treatment initiation and those changes are clinically relevant as they are predictive for further disease course (93). The reason for the faster improvement compared to CD is not entirely understood yet. It can be speculated that the submucosal oedema in UC rapidly resolves after effective treatment, which may explain this effect (94). This observation is supported by recent data from a monocenter study suggesting that the submucosa is the most response wall layer during treatment of active UC (94). In patients with CD, relevant changes with treatment response could be determined in a subgroup of patients as early as 4 weeks after treatment initiation (91). Therefore, evaluation after 4-8 weeks may already be useful in a subset of patients with highly active disease, not only in order to detect treatment response, but also in order to exclude any relevant complications or disease aggravation.

Based on the current data evaluation of FC and CRP after three months appears to be a reasonable time point to predict further disease course for most drugs. It can be speculated that a combination of different biomarkers might be beneficial for patient management. The use of IUS in combination with biomarkers to monitor disease activity has been suggested. However, data are controversial. In a recent study in UC, an additional benefit for using IUS plus calprotectin could not be shown (96), whereas another recent study demonstrated that the value of IUS is further enhanced when used in composite with FC.

Evaluation of disease course by IUS has shown that relevant parameters such as BWT and vascularization normalize in a relevant proportion of patients with CD already three months after treatment initiation (89). First results from the TRUST beyond trial show that IUS response in addition to CR is predictive for 1 year clinical outcome and superior to CR alone (97). Other preliminary results confirm these observations (98, 99).

Diagnostic algorithms may differ between UC and CD, as the time to induce various forms of remission are different between both disease entities. It also needs to be taken into account that various other factors determine the optimal time point of evaluation such as disease activity and disease severity. Early assessment of treatment response is more relevant in a patient with highly steroid refractory disease, compared to a patient with steroid dependent IBD. Different therapeutic agents vary in inducing remission and fast acting drugs such as JAK inhibitors require different time points of reassessment, compared to slower acting drugs such as anti-Integrins. In patients with CD, transmural response to treatment may also differ between ileum and colon, as recently demonstrated by IUS in patients treated with ustekinumab (91). The suggested algorithms therefore only provide a potential framework, which may need to be adapted in individual patients.

### Unmet needs and open research gaps

Further studies are required to establish diagnostic algorithms in monitoring patients with active IBD. For general implementation of IUS parameters as follow up, treat-to-target studies that include point-of-care IUS with central reading are mandatory. For those studies, validated ultrasound activity scores are necessary that show responsiveness to different treatment modalities and may predict clinical outcomes. In addition, studies are required that determine if IUS really leads to change in decision making, as well as cost-effectiveness studies demonstrating which diagnostic modality is best. Those studies do not only need to consider the cost of the individual diagnostic modality, but also the potential impact to change the disease course of the patients. Finally, best timepoints need to be defined for the use of IUS alone or in combination with other clinical, biochemical and other imaging modalities. Differences in disease severity, as well as differences in treatment modalities linked to response to given therapies, also need to be taken into account when diagnostic algorithms are validated.

As there is a growing interest in learning IUS worldwide, there is a need for a well-established training leading to competency. Despite the existence of training pathways offered by organisations such as IBUS, the training standards and defined competencies and their assessment require more formalized development (97, 98). Minimum standards for IUS examinations need to be defined not only to improve the quality but also to increase the rate of global acceptance for the use of IUS in IBD.

## Conclusion

During the last couple of years, treatment of IBD shifted away from purely symptom driven management. New diagnostic modalities for monitoring patients with IBD have been determined, such as IUS in combination with serum and faecal biomarkers that allow accurate, non-invasive, patient-centered and fast evaluation of disease activity and may predict disease course of individual patients. New diagnostic algorithms have been established to follow up of patients with active CD, as well as UC that guide treatment decisions. Even though clinical decisions involve a complex analysis of symptoms and the general patient’s condition, new diagnostic modalities such as IUS clearly help to improve management of patients with IBD. Individual diagnostic workup may still vary from the suggested algorithms, and may need to be adapted depending on the individual clinical scenario. Nevertheless, once suggested diagnostic algorithms have been confirmed in central-read RCTs, novel monitoring strategies may further reduce invasive endoscopy in the future.

## Author contributions

TK: writing manuscript, preparing figures BV: editing manuscript, preparing figures CM: editing manuscript, preparing figures. All authors contributed to the article and approved the submitted version.
